# Cardamonin Inhibited IL-1β Induced Injury by Inhibition of NLRP3 Inflammasome via Activating Nrf2/NQO-1 Signaling Pathway in Chondrocyte

**DOI:** 10.4014/jmb.2103.03057

**Published:** 2021-05-19

**Authors:** Jianqing Jiang, Mingsong Cai

**Affiliations:** No. 4 Trauma Area, Hangzhou Fuyang District Bone Injury Hospital of Traditional Chinese Medicine, Hangzhou City, Zhejiang Province, 311400, P.R. China

**Keywords:** Osteoarthritis, cardamonin, NLRP3 inflammasome, IL-1β, Nrf2/NQO-1

## Abstract

In this study we investigated the role and mechanism of cardamonin on IL-1β induced injury in OA. CHON-001 cells were treated with cardamonin and IL-1β and transfected with silencing nuclear factor erythroid 2-related factor 2 (siNrf2). Cell viability was detected by Cell Counting Kit-8 assay and flow cytometer assay was utilized for cell apoptosis assessment. IL-6, IL-8, TNF-α and Nrf2 mRNA expression was tested by qRT-PCR. Western blot was employed to evaluate MMP-3, MMP-13, Collagen II, Nrf2, NQO-1, NLRP3, Caspase 1 and apoptosis-associated speck-like protein containing a caspase-1 recruitment domain (ASC) protein levels. In CHON-001 cells, IL-1β suppressed cell viability and Collagen II level while promoting cell apoptosis and expression of pro-inflammatory cytokines (IL-6, IL-8, TNF-α), MMPs (MMP-3, MMP-13), NQO-1, and NLRP3 inflammasome (NLRP3, Caspase 1 and ASC), with no significant influence on Nrf2. Cardamonin reversed the effect of IL-1β on cell viability, cell apoptosis, pro-inflammatory cytokines, MMPs, Collagen II, and NLRP3 inflammasome levels. In addition, cardamonin advanced Nrf2 and NQO-1 expression of CHON-001 cells. SiNrf2 reversed the function of cardamonin on IL-1β-induced cell apoptosis and expression of pro-inflammatory cytokines, Nrf2, NQO-1, and NLRP3 inflammasome in chondrocytes. Taken together Cardamonin inhibited IL-1β induced injury by inhibition of NLRP3 inflammasome via activating Nrf2/NQO1 signaling pathway in chondrocyte.

## Introduction

Osteoarthritis (OA), a degenerative joint disease frequently connected with various factors including aging and obesity, is characterized by progressive destruction of joint structures such as subchondral bone and articular cartilage [1, 2]. OA affects about 300 million people around the world and its incidence is increasing in recent years due to aggravating trends such as an aging population and changes in living habits [3-6]. However, as a chronic disease with complex processes, unclear mechanisms and no effective therapy available, OA usually results in joint dysfunction and physical disability accompanied by long-term unbearable pain, placing a heavy burden on individuals, families and health care systems [7-10].

Accumulating evidence has demonstrated that inflammatory reaction, specifically inflammatory cytokines, plays a critical part in OA development [11, 12]. As one member of the inflammatory cytokine family, interleukin 1-beta (IL-1β), the elevated expression of which was found in synovial fluid and cartilage tissues of OA patients, is a pro-inflammatory cytokine reported to be involved in the pathophysiological process of OA through breaking the homeostatic balance via regulating cell factors in chondrocytes [12-16]. For example, IL-1β could induce chondrocytes to generate COX2 and iNOS, further facilitating the release of inflammatory mediators including prostaglandin E2 (PGE2) and nitric oxide (NO) [17]. Therefore, drugs against IL-1β may serve as a feasible treatment for OA.

Cardamonin is a chalcone isolated from the seed of a medicinal herb, *Alpinia katsumadai*, which has been extensively applied to treat digestive system-associated diseases such as emesis and gastric disorders [18, 19]. It is believed that cardamonin has a powerful effect on hypoglycemia, as well as vasodilation, antioxidation, anti-cancer and anti-inflammation activities [20]. Although the role of cardamonin in OA and its mechanism of action still remain largely unknown, previous studies have revealed that cardamonin could exert its anti-inflammatory function via suppressing activation of NF-κB and MAPK signaling pathways [21-24]. What’s more, cardamonin resists LPS-induced septic shock and attenuates inflammatory bowel disease through repressing nucleotide binding oligomerization domain-like receptor 3 (NLRP3) inflammasome [19, 25]. NLRP3 inflammasome, as a key element in inflammatory response, was proved to be implicated in OA advancement as the inhibition of NLRP3 inflammasome mitigated OA [26-28]. Hence, we presumed that cardamonin might affect OA progression through suppressing NLRP3 inflammasome activation.

In our research, we began by investigating the role of cardamonin in IL-1β-mediated chondrocytes. Then we probed further into its specific mechanism, attempting to gain a better understanding of OA processes and mechanisms as well as to offer a novel and effective agent for OA treatment.

## Materials and Methods

### Cell Culture

The human chondrocyte line CHON-001 was bought from American Type Culture Collection (ATCC; ATCC CRL-2846, USA). Cells were cultured in Dulbeccós Modified Eaglés Medium (DMEM; ATCC 30-2002, USA) supplemented with 0.1 mg/ml G-418 (G8160, Beijing Solarbio Science & Technology Co., Ltd., China) and 10%heat-inactivated fetal bovine serum (FBS; 10100147, Gibco, Thermo Fisher Scientific, USA) in a humidified incubation at 37°C with 5% CO_2_.

### Agent Treatment

To explore the role of cardamonin in chondrocytes, cells were treated with different concentrations of cardamonin (C16H14O4, ≥98.0%, 1, 3, 10, 30, 100 μmol/l, C8249, Sigma-Aldrich, USA) for 24 h. During the experiments on the function of cardamonin on IL-1β-mediated cells, CHON-001 cells were exposed to 10 ng/ml IL-1β for 12 h, followed with different doses of cardamonin (1, 3, 10, 30 μmol/l) for 24 h in Cell Counting Kit-8 (CCK-8) assay. And for a better experimental effect, treatment of 10 ng/ml IL-1β with 10, 30 μmol/l cardamonin was adopted for later experiments in this area. In the research on the mechanism of cardamonin, cells were treated with 10 ng/ml IL-1β alone or in combination with 30 μmol/l cardamonin.

### Cell Transfection

Silencing Nrf2 (siNrf2; siB151231100700-1-5, 5’-AGCAAUUGCGCAACAGAUCAAGAUCUGUUGCGC AAUUGCUAU-3’, Guangzhou RiboBio Co., Ltd., China) and its negative control (siNC; siN0000001-1-10, Guangzhou RiboBio Co., Ltd.) were transfected into cells. LipoRNAi Transfection Reagent (C0535, Beyotime Biotechnology, China) was employed for cell transfection. Briefly, cells were trypsinized (T1350, Beijing Solarbio Science & Technology Co., Ltd.) and seeded at 2 × 10^5^ cells/well in 6-well plates, and then cultured until 70-80%confluence. SiNrf2 or siNC (100 pmol) was diluted in Opti-MEM medium (125 μl; 31985062, Thermo Fisher Scientific) and mixed well. Then 4 μl LipoRNAi Transfection Reagent was added and gently mixed again, following which, cells were cultured at room temperature for 20 min. Finally, the mixture (125 μl/well) was added to cells and the plates were incubated for 24 or 48 h at 37°C.

### Cell Counting Kit-8 (CCK-8) Assay

Cell viability was detected through CCK-8 assay. With transfection and agent treatment, cells in the logarithmic growth were trypsinized and seeded in 96-well plates at a density of 1 × 10^4^ cells/well, followed by culture with 5% CO_2_ at 37°C for 24 h. Subsequently, 10 μL CCK-8 solution (CA1210, Beijing Solarbio Science & Technology Co., Ltd.) was added into each well and cells were incubated for another 4 h at 37°C. The absorbance was measured at 450nm by a microplate reader (SpectraMax iD5, Molecular Devices, USA).

### Flow Cytometer Assay

Cell apoptosis was assessed by Annexin V-FITC Apoptosis Detection Kit (C1062S, Beyotime Biotechnology, China). After transfection and agent treatment, cells were trypsinized and rinsed with PBS, subsequent to which cells were centrifuged at 1,000 ×*g* for 5 min. Supernatant discarded, cells were harvested and suspended in PBS. Cells (5×10^4^) were centrifuged at 1,000 ×*g* for 5 min again and resuspended in 195 μl Annexin V-FITC binding buffer after removal of supernatant. Annexin V-FITC (5 μl) and PI (10 μl) were successively added to cells and mixed well. Cells were incubated at room temperature for 15 min in a dark room. The flow cytometer (CytoFLEX, Beckman Coulter, Inc., USA) was adopted for analysis of cell apoptosis.

### Quantitative Reverse Transcription-Polymerase Chain Reaction (qRT-PCR)

Trizol Reagent (R0016, Beyotime Biotechnology, China) was utilized to isolate total mRNA from cells. Hifair III 1st Strand cDNA Synthesis SuperMix for qPCR (gDNA digester plus) (11141ES10, Yeasen Biotech Co., Ltd., China, http://www.yeasenbiotech.com/) was applied to synthesize cDNA through reverse transcription, followed by amplification of cDNA in an ABI 7000 real-time fluorescence quantitative PCR instrument (Thermo Fisher Scientific), with Hieff UNICON Universal Blue qPCR SYBR Green Master Mix (11184ES03, Yeasen Biotech Co., Ltd., China) tracing the quantitative PCR of mRNA under condition of a thermal cycling program: predenaturation at 95°C for 30 s, followed with 40 cycles of 95°C for 3 s and 60°C for 20 s. Primer sequences (Guangzhou RiboBio Co., Ltd., China) were listed in Table 1. β-Actin was presented as an endogenous control. Data were calculated using the 2^-ΔΔCT^ relative quantification method [29].

### Western Blot

Total protein was extracted by radio immunoprecipitation assay (RIPA) lysis buffer (P0013E, Beyotime Biotechnology) and centrifuged at 14,000 ×*g* at 4°C for 3 min. Supernatant was collected and total protein concentration was evaluated by BCA Protein Assay Kit (P0012S, Beyotime Biotechnology). Equal contents of protein and ColorMixed Protein Marker (11-180KD) (PR1910, Beijing Solarbio Science&Technology Co., Ltd.) were separated through a 6%-10% SDS polyacrylamide gel electrophoresis (SDS-PAGE) preparation kit (C631100, Sangon Biotech Co., Ltd., China), following which, mixture was transferred to PVDF membranes (88585, Thermo Fisher Scientific) blocked in 5% bovine serum albumin (BSA; PC0001, Beijing Solarbio Science & Technology Co., Ltd.) for 1 h and then incubated at 4°C with primary antibodies including rabbit anti-NAD(P)H quinone dehydrogenase 1 (NQO-1) (1:1000; #62262, CST, USA), rabbit anti-NLRP3 (1:1000; ab263899, Abcam, Cambridge, USA), rabbit anti-matrix metalloproteinase (MMP)-13 (1:6000; ab39012, Abcam), rabbit anti-MMP-3 (1:20000; ab52915, Abcam), rabbit anti-Caspase 1 (1:200; ab138483, Abcam, USA), rabbit anti-Collagen II (1:10000; ab188570, Abcam), rabbit anti-ASC (1:10000; ab151700, Abcam), mouse anti-β-actin (1:1000; ab8226, Abcam) and rabbit anti-Nrf2 (1:1000; ab62352, Abcam). After one night, membranes were washed with Tris-buffered saline containing Tween 20 (TBST; BI-WB027, Nanjing SenBeiJia Biological Technology Co., Ltd., China, http://www.senbeijia.com/), followed by incubation with the corresponding secondary antibodies anti-rabbit IgG H&L (ab6721, 1:5000; Abcam) and anti-mouse IgG H&L (ab6728, 1:5000; Abcam) for 1 h at room temperature and washing five times with TBST for 5 min. The proteins were visualized using electrochemiluminescence (ECL) reagent (PE0010, Beijing Solarbio Science & Technology Co., Ltd.) through the SH-Focus523 Chemiluminescence System (Shenhua Bio. Co., Ltd., China, http://www.shenhuabio.cn/index.php), as Image J software version 1.48 (National Institutes of Health, USA) was applied for analysis.

### Statistical Analysis

All experiments were repeated independently at least three times. Statistical analysis was conducted by GraphPad Prism 8.0 (GraphPad Software Inc., USA) and SPSS 20.0 software (SPSS Inc., USA). Data were performed as the means ± SD. The differences between multiple groups was analyzed by one-way ANOVA and followed by Tukey post hoc test. A statistically significant difference was accepted when *p* < 0.05.

## Results

### Cardamonin Reversed the Effect of IL-1β on Repressing Viability and Inducing Apoptosis as well as Inflammatory Factor Levels in Chondrocytes

**Fig. 1:**
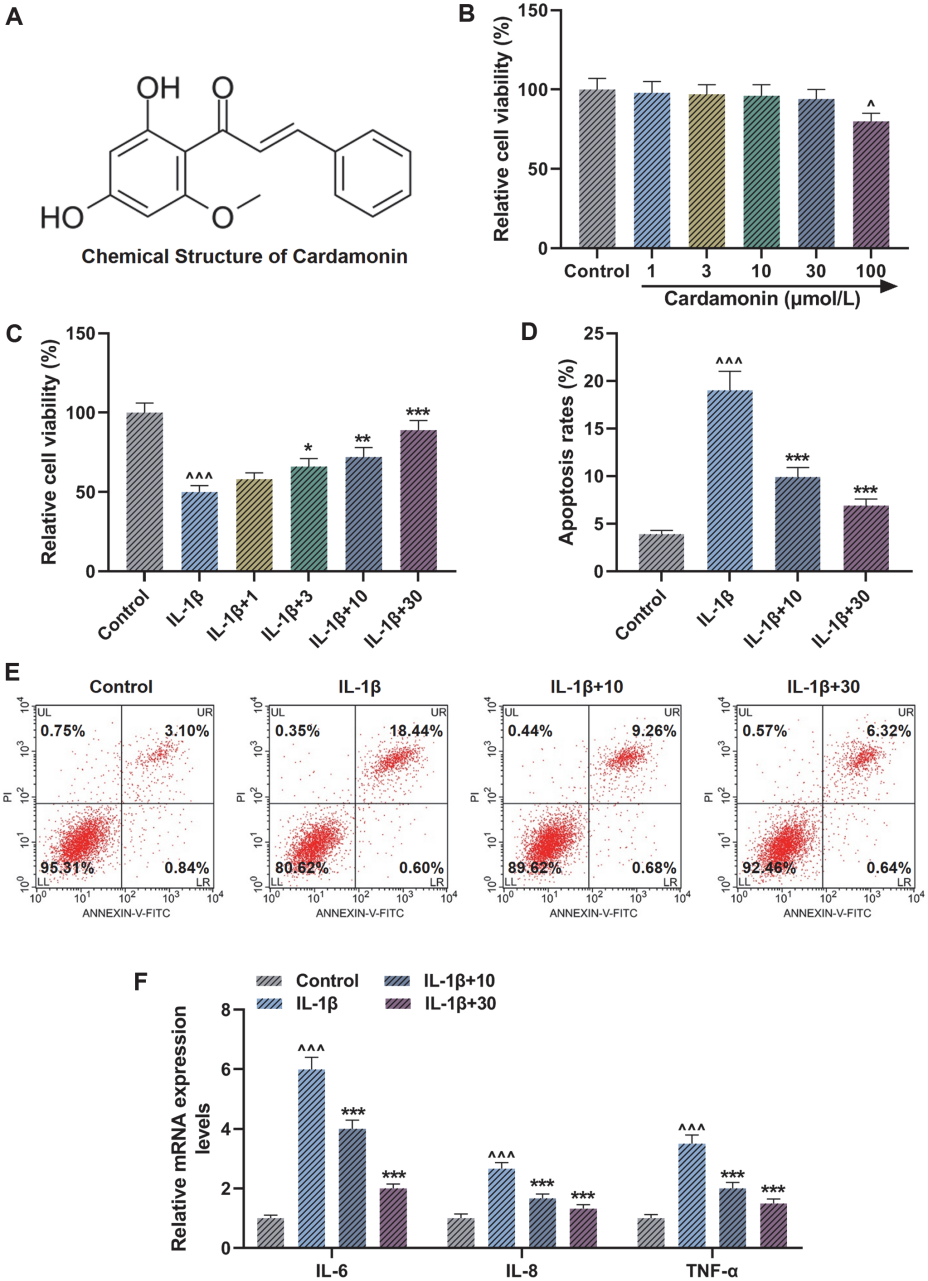
Cardamonin reversed the effect of interleukin (IL)-1β on repressing viability and inducing apoptosis as well as inflammatory factor levels in chondrocytes. (**A**) Chemical structure of cardamonin. (**B** and **C**) Cell viability was detected by Cell Counting Kit-8 (CCK-8) assay after treatment of different cardamonin concentrations (**B**) as well as treatment of IL-1β in combination with cardamonin (**C**). (**D**) Cell apoptosis was tested through flow cytometer assay after treatment of IL-1β and cardamonin. (**E**) Representative images of cell apoptosis tested by flow cytometer assay after treatment of IL-1β and cardamonin. (**F**) IL-6, IL-8 and tumor necrosis factor (TNF)-α mRNA levels were assessed by quantitative reverse transcription-polymerase chain reaction (qRT-PCR) after treatment of IL-1β and cardamonin. ^^^*p* < 0.001 vs. Control group; ^*p* < 0.05 vs. Control group; ***p < 0.001 vs. IL-1β group; **p < 0.01 vs. IL-1β group; *p < 0.05 vs. IL-1β group. All experiments were repeated independently at least three times. Data were performed as the means ± SD.

The chemical structure of cardamonin was presented in Fig. 1A. During the experiment on detecting cytotoxicity of cardamonin in human normal chondrocytes, we found that there was no significant difference in cell viability among treatment with 1, 3, 10, 30 μmol/l cardamonin but 100 μmol/l cardamonin obviously inhibited cell viability (Fig. 1B, *p* < 0.05), indicating that 100 μmol/l cardamonin had cytotoxicity on CHON-001 cells. Thus, we chose 1, 3, 10, 30 μmol/l cardamonin for later research. The CCK-8 assay results revealed that CHON-001 cells treated with IL-1β decreased cell viability in comparison with Control group (Fig. 1C, *p* < 0.001) while the co-treatment of IL-1β and cardamonin increased cell viability in a dose-dependent manner when compared with IL-1β group (Fig. 1C, *p* < 0.05). Furthermore, owing to the more prominent effect presented in treatment of 10 and 30 μmol/l cardamonin, we selected those two cardamonin concentrations for subsequent experiments. In addition, in contrast with Control group, a marked increase of cell apoptosis rate was observed in IL-1β group (Figs. 1D and 1E, *p* < 0.001). In contrast with cells treated with IL-1β alone, cells treated with IL-1β and cardamonin decreased the apoptosis rate as the cardamonin dose rose (Figs. 1D and 1E, *p* < 0.001). A similar consequence was also obtained in the assessment of IL-6, IL-8, and tumor necrosis factor (TNF)-α mRNA expression (Fig. 1F, *p* < 0.001), indicating cardamonin could partly offset the IL-1β-induced inflammatory reaction in human chondrocytes.

### Cardamonin Reversed the IL-1β-Induced Expression of Collagen-Related Factors and Activation of NLRP3 Inflammasome in Chondrocytes

**Fig. 2:**
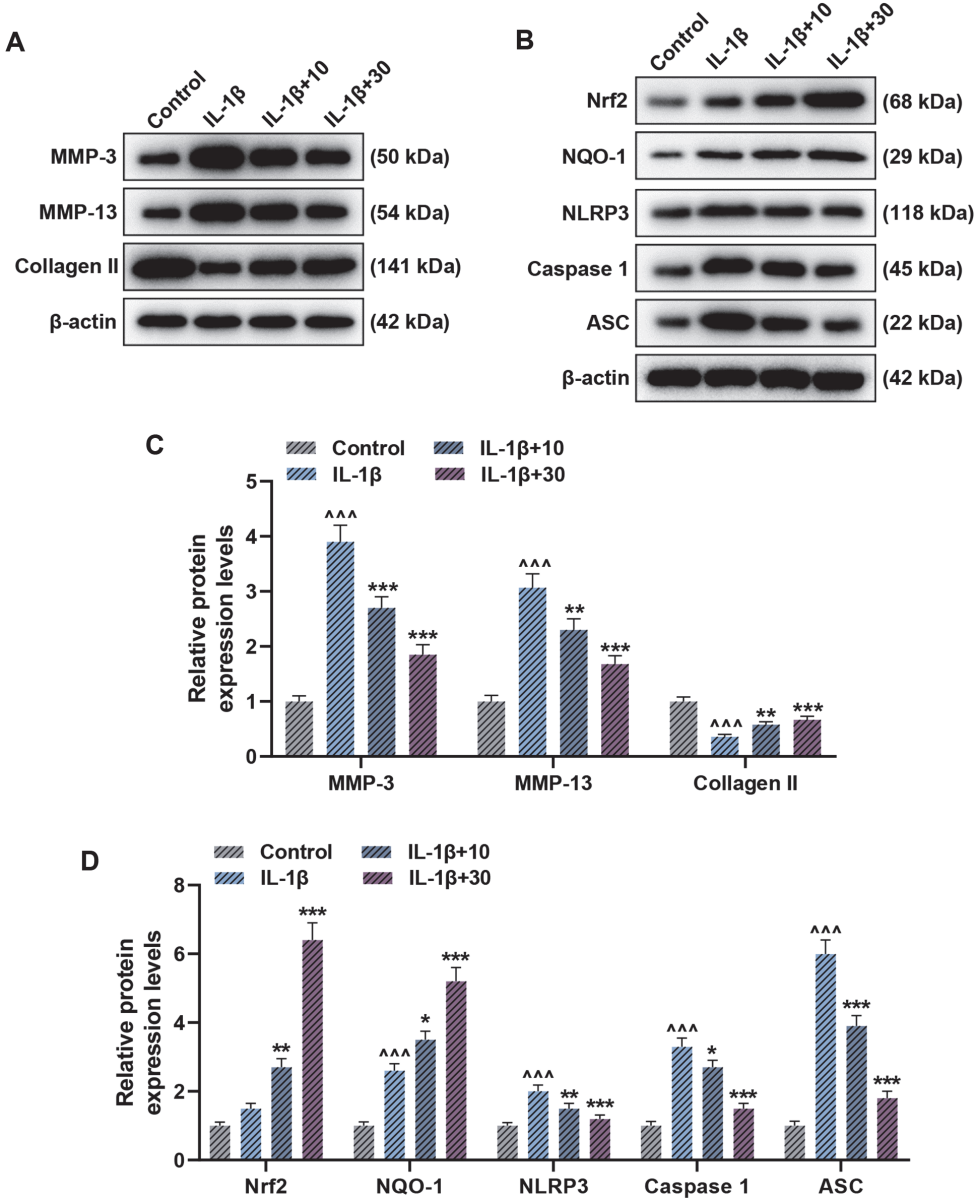
Cardamonin reversed the interleukin (IL)-1β-induced expression of collagen-related factors and activation of nucleotide binding oligomerization domain-like receptor 3 (NLRP3) inflammasome in chondrocytes. (**A** and **B**) Representative images of matrix metalloproteinase (MMP)-3, MMP-13 and Collagen II (**A**) as well as nuclear factor erythroid 2-related factor 2 (Nrf2), NAD(P)H quinone dehydrogenase 1 (NQO-1), NLRP3, Caspase 1 and ASC (**B**) protein expression detected by western blot after treatment of IL-1β and cardamonin. β-Actin was used as a loading control. (**C** and **D**) MMP-3, MMP-13 and Collagen II (**C**) as well as Nrf2, NQO-1, NLRP3, Caspase 1 and ASC (**D**) protein expression was detected by western blot after treatment of IL-1β and cardamonin. β-Actin was used as a loading control. ^^^*p* < 0.001 vs. Control group; ***p < 0.001 vs. IL-1β group; **p < 0.01 vs. IL-1β group; *p < 0.05 vs. IL-1β group. All experiments were repeated independently at least three times. Data were performed as the means ± SD.

Through the western blot, it was discovered that compared with Control group, CHON-001 cells in IL-1β group elevated the MMP-3, MMP-13, NQO-1, NLRP3, Caspase 1 and ASC levels while decreasing Collagen II expression obviously, with no apparent effect on Nrf2 viewed (Figs. 2A-2D, *p* < 0.001). In contrast with IL-1β group, cardmonin reduced MMP-3, MMP-13, NLRP3, Caspase 1 and ASC expression while increasing Collagen II, Nrf2, and NQO-1 protein levels in a dose-dependent manner (Figs. 2A-2D, *p* < 0.05), implying that the effect of cardamonin on IL-1β-induced NLRP3 inflammasome activation in chondrocytes might be related to Nrf2/NQO-1 signaling pathway.

### SiNrf2 Reversed the Effect of Cardamonin on Nrf2 Expression and Cell Apoptosis in IL-1β-Mediated Chondrocytes

**Fig. 3:**
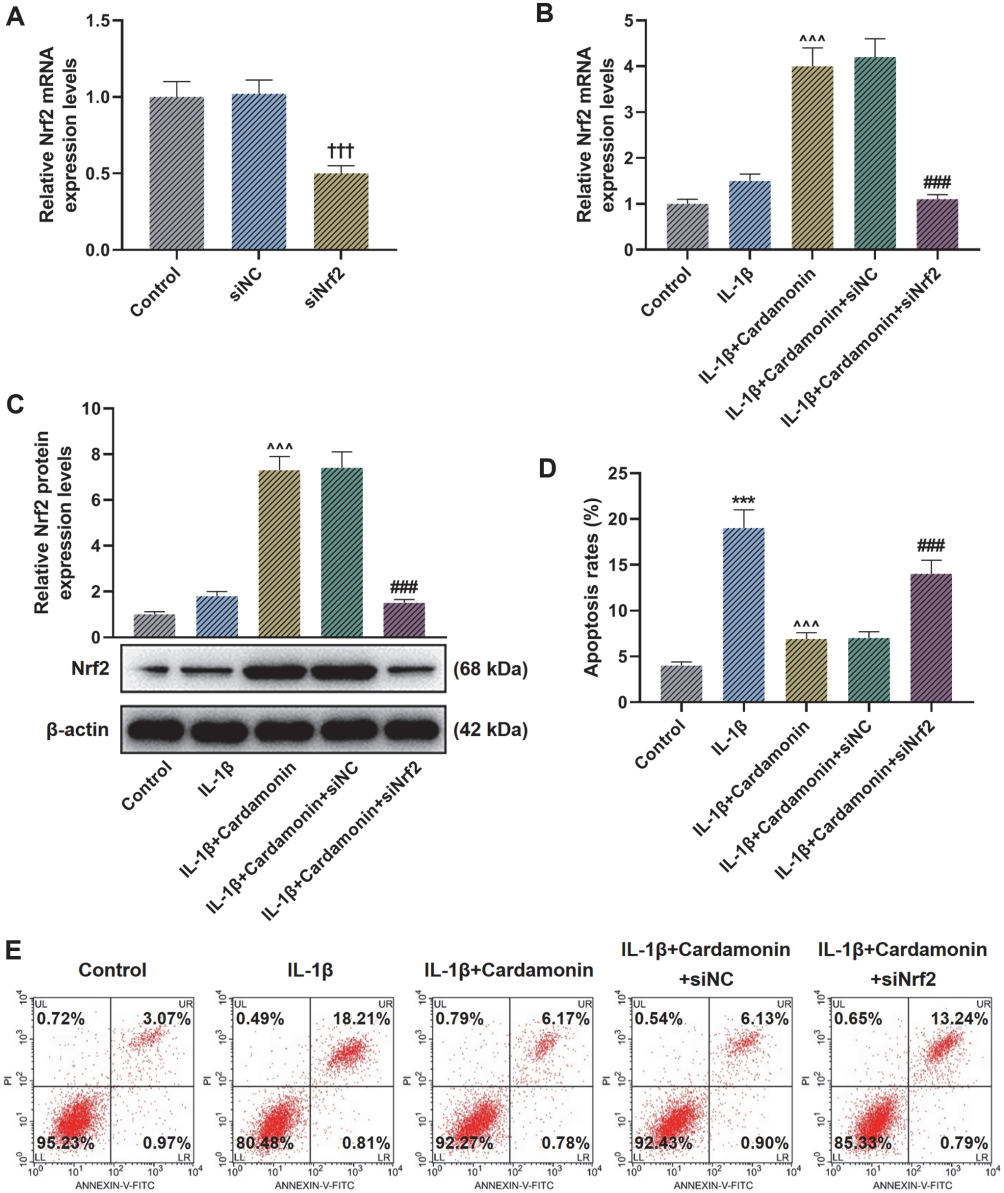
Silencing nuclear factor erythroid 2-related factor 2 (siNrf2) reversed the effect of cardamonin on Nrf2 expression and cell apoptosis in interleukin (IL)-1β-mediated chondrocytes. (**A** and **B**) Relative Nrf2 mRNA expression was tested through quantitative reverse transcription-polymerase chain reaction (qRT-PCR) after transfection of siNrf2 without (**A**) or with (**B**) treatment of IL-1β and cardamonin. (**C**) Relative Nrf2 protein expression was assessed by western blot after treatment of IL-1β and cardamonin as well as transfection of siNrf2. β-Actin was used as a loading control. (**D**) Cell apoptosis was tested through flow cytometer assay after treatment of IL-1β and cardamonin as well as transfection of siNrf2. (**E**) Representative images of cell apoptosis tested by flow cytometer assay after treatment of IL-1β and cardamonin as well as transfection of siNrf2. ^†††^*p* < 0.001 vs. silencing negative control (siNC) group; ***p < 0.001 vs. Control group; ^^^*p* < 0.001 vs. IL-1β group; ###*p* < 0.001 vs. IL-1β+Cardamonin+siNC group. All experiments were repeated independently at least three times. Data were performed as the means ± SD.

Due to a better and more prominent effect performed in treatment of 30 μmol/l cardamonin, we chose that dose for the following research. The qRT-PCR results showed that cells transfected with siNrf2 markedly lowered the Nrf2 mRNA level when contrasted with siNC group (Fig. 3A, *p* < 0.001), indicating that the Nrf2 silencing model was successfully established. Moreover, as no significant effect was observed between Control group and IL-1β group, CHON-001 cells treated with IL-1β in combination with cardamonin showed elevated Nrf2 expression in comparison with cells treated with IL-1β alone (Figs. 3B and 3C, *p* < 0.001). Meanwhile, Nrf2 level in IL-1β+Cardamonin+siNrf2 group was much lower than IL-1β+Cardamonin+siNC group (Figs. 3B and 3C, *p* < 0.001). Additionally, the findings of the cell apoptosis evaluation revealed that the apoptosis rate in IL-1β group was higher than Control group (Figs. 3D and 3E, *p* < 0.001). In addition, contrary to the results of Nrf2 expression detection, cells in IL-1β+Cardamonin group decreased the apoptosis rate in comparison with IL-1β group (Figs. 3D and 3E, *p* < 0.001) while IL-1β+Cardamonin+siNrf2 group increased the CHON-001 cell apoptosis rate in contrast with IL-1β+Cardamonin+siNC group (Figs. 3D and 3E, *p* < 0.001).

### SiNrf2 Reversed the Effect of Cardamonin on IL-1β-Induced Expression of Inflammatory Factors and Activation of NLRP3 Inflammasome in Chondrocytes

**Fig. 4:**
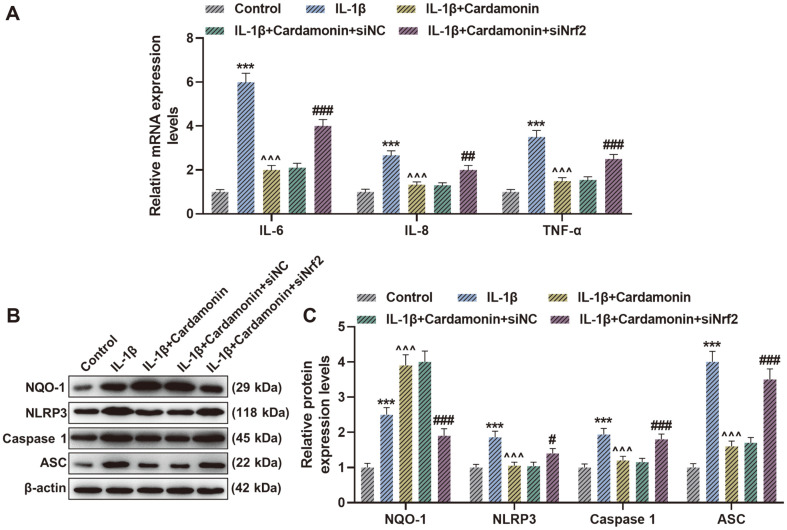
Silencing nuclear factor erythroid 2-related factor 2 (siNrf2) reversed the effect of cardamonin on interleukin (IL)-1β-induced expression of inflammatory factors and activation of nucleotide binding oligomerization domain-like receptor 3 (NLRP3 ) inflammasome in chondrocytes. (**A**) IL-6, IL-8 and tumor necrosis factor (TNF)-α mRNA expression levels were assessed by quantitative reverse transcription-polymerase chain reaction (qRT-PCR) after treatment of IL-1β and cardamonin as well as transfection of siNrf2. (**B** and **C**) Representative images of NAD(P)H quinone dehydrogenase 1 (NQO-1), NLRP3, Caspase 1 and ASC protein bands (**B**) and NQO-1, NLRP3, Caspase 1 and ASC protein expression levels (**C**) were evaluated through western blot after treatment of IL-1β and cardamonin as well as transfection of siNrf2. β-Actin was used as a loading control. ***p < 0.001 vs. Control group; ^^^*p* < 0.001 vs. IL-1β group; ^###^*p* < 0.001 vs. IL-1β+Cardamonin+silencing negative control (siNC) group; ^##^*p* < 0.01 vs. IL-1β+Cardamonin+siNC group; ^#^*p* < 0.05 vs. IL-1β+Cardamonin+siNC group. All experiments were repeated independently at least three times. Data were performed as the means ± SD.

As IL-1β group presented higher expression of IL-6, IL-8, TNF-α, NQO-1, NLRP3, Caspase 1 and ASC than Control group (Figs. 4A-4C, *p* < 0.001), IL-1β+Cardamonin group showed reduced IL-6, IL-8, TNF-α, NLRP3, Caspase 1 and ASC levels and elevated NQO-1 when contrasted with IL-1β group (Figs. 4A-4C, *p* < 0.001). However, the opposite consequence was observed in IL-1β+Cardamonin+siNrf2 group in comparison with IL-1β+Cardamonin+siNC group (Figs. 4A-4C, *p* < 0.05). The results suggested that cardamonin inhibited IL-1β-induced increased expression of inflammatory factors and NLRP3 inflammasome activation in chondrocytes via Nrf2/NQO1 signaling pathway.

## Discussion

As one of the most common forms of arthritis, with no efficient therapy as well as gradually elevating morbidity, OA is a heterogeneous and prevalent condition among the elderly, adding a tremendous burden to society [30-32]. Cardamonin is a biological active extract from seeds of *Alpinia katsumadai* with anti-inflammatory effect [18-20]. However, the function of cardamonin and how cardamonin affects OA have not been expounded. Due to reports about cardamonin repressing NLRP3 inflammasome and OA alleviated by inhibition of NLRP3 inflammasome [19, 27, 28], we supposed that cardamonin might have an impact on OA via NLRP3 inflammasome.

Firstly, a test of cell viability was applied to determine the effect of cardamonin on CHON-001 cells, the consequence of which showed that 100 μmol/l cardamonin had cytotoxicity to human normal chondrocytes, narrowing down the choice range of optimal dose in the treatment of OA.

IL-1β, an inflammatory cytokine whose upregulation positively participates in OA progression, has been widely adopted as an inducer of OA in vitro [12, 14, 33]. Therefore, we used IL-1β for simulation of OA pathophysiology in our study.

Secondly, we investigated the role of cardamonin in OA. The results of CCK-8 and flow cytometer assays revealed that cardamonin reversed the effect of IL-1β on inhibiting viability and advancing apoptosis of chondrocytes in a dose-dependent manner, which implied that cardamonin fulfilled a protective function against IL-1β-induced injury in chondrocytes.

IL-6, IL-8 and tumor necrosis factor-α (TNF-α) are all key pro-inflammatory cytokines triggering robust inflammatory response, the high expression levels of which were discovered in the joint fluid and serum of OA [10, 25, 34]. In agreement with previous research on the effect of ligustrazine and lycium barbarum polysaccharide on chondrocytes mediated by IL-1β, our study observed that IL-1β stimulation promoted IL-6, IL-8 and TNF-α expression while cardamonin treatment partly decreased the upregulation of those inflammatory factors induced by IL-1β, suggesting that cardamonin might serve as an anti-inflammatory drug in OA.

Belonging to the group functioning to degrade the extracellular matrix (ECM), MMPs play an important role in OA development, among which MMP-3 and MMP-13 are essential elements that degrade the cartilage matrix [35-37]. Collagen II is also one of the major components making up the cartilage matrix [38]. Through western blot, it was reported that cardamonin reversed the IL-1β-induced elevation of MMP-3 as well as MMP-13 levels and reduction of Collagen II expression, which indicated that cardamonin protected chondrocytes from cartilage matrix degradation in OA.

Nrf2 is a redox-sensitive transcription factor of the leucine zipper family, regarded as having a vital role in participating in modulation on cell reaction to oxidative stresses [39]. Lots of studies have demonstrated that Nrf2 certainly contributes to defending tissues against chronic inflammation and malignancies through resisting oxidative stress [40, 41]. Moreover, NQO1 is well known as a responsive antioxidant gene of Nrf2 [25]. Also playing a significant part in inflammatory responses, the NLRP3 inflammasome is a multiprotein complex composed of NLRP3, ASC and Caspase-1 [19]. Previous research has shown that Nrf2 activation mitigated IL-1β-induced matrix degradation of chondrocytes and cardamonin could inhibit the NLRP3 inflammasome through activating Nrf2/NQO1 signaling pathway [19, 25, 39]. Nrf2 activation negatively regulates caspase-1 cleavage, IL-1β maturation and priming process of NLRP3 inflammasome by upregulating NQO1 [42]. IL-1β stimulation of human chondrocytes led to a significant upregulation of the expression of Nrf2 and NQO1 [39]. Similarly, in our experiments, cardamonin prominently increased Nrf2 and NQO-1 expression while reversing the upregulation of NLRP3, ASC and Caspase-1 caused by IL-1β, indicating that the negative effect of cardamonin on OA advancement might relate to Nrf2/NQO1 signaling pathway.

Thirdly, we explored the mechanism of cardamonin on OA. An Nrf2 silencing model was employed to inversely validate our assumption. The consequence that an obvious decline of Nrf2 expression was viewed after transfection of siNrf2 indicated the successful establishment of a gene silencing model. Moreover, qRT-PCR, flow cytometer and western blot showed that siNrf2 reversed the effect of cardamonin on ameliorating IL-1β-induced cell apoptosis and cell factor expression in chondrocytes, which suggested that cardamonin exerted its protective effect against OA via activating Nrf2.

Taking the above all together, our study elucidated cardamonin's defensive role against OA by inhibition of NLRP3 inflammasome via activating Nrf2/NQO1 signaling pathway, providing a potential agent for OA treatment. Nevertheless, further experiments into the effect of cardamonin in vivo should be performed in the future to verify the conclusion of this thesis. We will also continue to probe into the targets and mechanisms of cardamonin in OA.

All in all, this paper attested that cardamonin inhibited IL-1β-induced injury by inhibition of NLRP3 inflammasome via activating Nrf2/NQO1 signaling pathway, implying that cardamonin might perform as a promising therapy choice for improving management of OA. But more research and clinical trials are needed to further clarify the efficacy of cardamonin on OA.

## Figures and Tables

**Table 1:** Primer sequences used for quantitative reverse transcription-polymerase chain reaction (qRT-PCR).

Target gene	Primers, 5’-3’
IL-6	
(Forward)	ACTCACCTCTTCAGAACGAATTG
(Reverse)	CCATCTTTGGAAGGTTCAGGTTG
IL-8	
(Forward)	GTGCATAAAGACATACTCCA
(Reverse)	CTCTTCAAAAACTTCTCCAC
TNF-α	
(Forward)	CCTCTCTCTAATCAGCCCTCTG
(Reverse)	GAGGACCTGGGAGTAGATGAG
Nrf2	
(Forward)	TCAGCGACGGAAAGAGTATGA
(Reverse)	CCACTGGTTTCTGACTGGATGT
β-actin	
(Forward)	CTCCATCCTGGCCTCGCTGT
(Reverse)	GCTGTCACCTTCACCGTTCC
